# Engineered collagen polymeric materials create noninflammatory regenerative microenvironments that avoid classical foreign body responses†

**DOI:** 10.1039/d3bm00091e

**Published:** 2023-03-21

**Authors:** Rachel A. Morrison, Sarah Brookes, Theodore J. Puls, Abigail Cox, Hongyu Gao, Yunlong Liu, Sherry L. Voytik-Harbin

**Affiliations:** a Weldon School of Biomedical Engineering, Purdue University West Lafayette IN 47907 USA harbins@purdue.edu; b GeniPhys Inc. Zionsville IN 46077 USA; c Department of Comparative Pathobiology, Purdue University West Lafayette IN 47907 USA; d Department of Medical & Molecular Genetics, Indiana University School of Medicine Indianapolis IN 46202 USA; e Department of Basic Medical Sciences, Purdue University West Lafayette IN 47907 USA

## Abstract

The efficacy and longevity of medical implants and devices is largely determined by the host immune response, which extends along a continuum from pro-inflammatory/pro-fibrotic to anti-inflammatory/pro-regenerative. Using a rat subcutaneous implantation model, along with histological and transcriptomics analyses, we characterized the tissue response to a collagen polymeric scaffold fabricated from polymerizable type I oligomeric collagen (Oligomer) in comparison to commercial synthetic and collagen-based products. In contrast to commercial biomaterials, no evidence of an immune-mediated foreign body reaction, fibrosis, or bioresorption was observed with Oligomer scaffolds for beyond 60 days. Oligomer scaffolds were noninflammatory, eliciting minimal innate inflammation and immune cell accumulation similar to sham surgical controls. Genes associated with Th2 and regulatory T cells were instead upregulated, implying a novel pathway to immune tolerance and regenerative remodeling for biomaterials.

## Abstract 2

Engineered collagen polymeric scaffolds exhibit regenerative remodeling without eliciting a foreign body reaction, fibrosis, or bioresorption.

## Graphical abstract

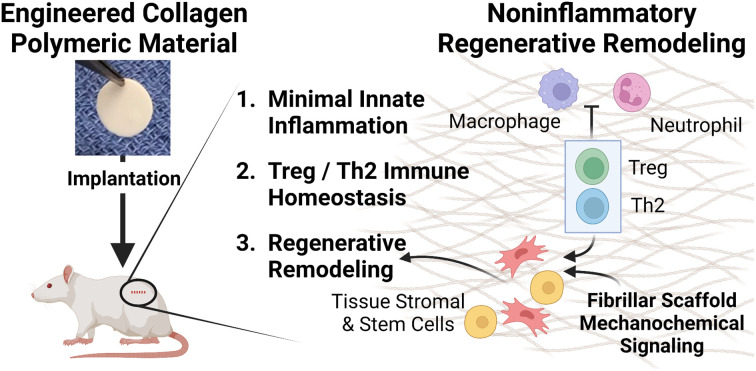

## 1. Introduction

Biomaterials play an integral role in modern health care, with applications extending from hemostatic and wound dressings to medical implants for tissue reinforcement or replacement (*e.g.*, hernia mesh, vascular graft, breast implant, artificial joints), to various medical devices (*e.g.*, artificial pancreas, pacemaker). By definition, a biomaterial is any substance (other than a drug), synthetic or natural, that can be used as a system or part of a system that treats, augments, or replaces any tissue, organ, or function of the body.^1^ This definition immediately brings to light the importance of biomaterial-tissue interactions and associated tissue responses as prioritized considerations when designing biomaterials for various intended uses. Synthetic materials, whether permanent or degradable, continue to be attractive candidates for medical product design, owing to advantages associated with sourcing, batch-to-batch reproducibility, high customizability, physical stability, amenability to scalable manufacturing, and cost. However, increasing concerns have been raised regarding fibrotic encapsulation and associated foreign body reactions (FBRs) elicited by these materials as well as their lack of biological signaling capacity (bioactivity).^2,3^ Alternative approaches have targeted use or incorporation of biological materials, including collagen, glycosaminoglycans, and other components of the tissue extracellular matrix (ECM). Here, the goal is to engage the body's cells *via* binding and signaling motifs inherent to these biomolecules and their structural assemblies for improved material biocompatibility and tissue integration.^4^ While biological materials are routinely used for ound, dental, and various surgical applications, their broader utility is limited by sensitivity to standard manufacturing processes, limited customizability of material format and physical properties, and rapid immune-mediated material degradation (bioresorption).^4–6^ Consequently, the search continues for advanced engineering biomaterials that are highly customizable and adaptable to patient-specific needs and support improved tissue response outcomes, namely promotion of immune tolerance, tissue integration, and stimulation of scar-free regenerative healing, where compromised tissues are restored to a normal histological and functional state.^7–9^

It is well established that nature-derived or synthetic materials not inherent to the host are recognized as foreign objects by the immune system. Also recognized as foreign are remnant cellular debris and modified biomolecules (*e.g.*, denatured, chemically modified), which are often associated with tissue-derived or other biological materials. Implantation of these foreign materials yields well-characterized FBRs, which are initiated by the immune system at the tissue-implant interface.^10–14^ Mechanistically, FBRs are often described as having multiple sequential but overlapping phases, including protein adsorption, acute inflammation, chronic inflammation, foreign body giant cell (FBGC) formation, and fibrosis/fibrous capsule formation.^2,15^ This reaction initiates immediately upon tissue contact, with blood-derived and interstitial protein adsorption to the material surface and associated complement activation (the first step of innate immunity). In turn, chemokines and other chemoattractants guide cells of the innate or non-specific immune system, notably neutrophils, mast cells, dendritic cells, monocytes, and macrophages, to accumulate at the implant site. Continued activation of macrophages, as well as their fusion to form multi-nucleated FBGCs, leads to a state of chronic inflammation, with engagement of adaptive immune cell populations (*e.g.*, lymphocytes including cytotoxic T cells, T helper cells, and B cells), recruitment of additional cell types (*e.g.*, fibroblasts, myofibroblasts), and ultimately deposition of dense fibrous connective tissue (fibrotic capsule) that walls off the foreign material. This process is further exacerbated by phagocytosis, where neutrophils, macrophages, and FBGCs work to actively engulf and break down foreign materials, releasing reactive oxygen species and matrix degradative enzymes.

While FBGCs, fibrosis, and fibrous capsule formation are commonly reported outcomes following biomaterial implantation, immune cell phenotypes and cellular signaling pathways, material degradation or bioresorption, thickness of the fibrous capsule, and consequences of the inflammatory response vary with biomaterial type and implantation microenvironment.^16^ Early mechanistic studies focusing on the innate immune system showed that macrophage polarization and phenotype play a major role in determining biomaterial FBRs and outcomes.^17^ However, growing evidence now suggests that biomaterial tissue reaction initiation and resolution are driven by crosstalk between both innate and adaptive immune cell players. More specifically, macrophages, depending upon their phenotype and cytokine/chemokine profile, recruit and differentially engage CD8+ cytotoxic T cells and various CD4+ T helper cells.^18–20^ Although T helper cells are activated through antigen presentation by macrophages and dendritic cells, the biomaterial microenvironment greatly influences subtype, including differentiation along pro-inflammatory Th1/Th17 pathways or Th2/regulatory T (Treg) pathways that suppress inflammation and promote immune homeostasis.^21^ Given that both innate and adaptive immune cells operate along phenotypic continuums extending from pro-inflammatory/pro-fibrotic to anti-inflammatory/pro-regenerative, it is this delicate balance that ultimately determines biomaterial tissue response and healing outcomes.^21^ To influence this balance, many current biomaterial design strategies focus on modulating the tissue response by manipulating specific physicochemical properties, including size and shape,^22^ pore size,^23^ surface properties (*e.g.*, hydrophilicity,^24^ surface charge,^25^ chemical functionalization^26,27^), mechanical properties,^28,29^ and degradability.^30^ To further augment biomaterials and promote favorable host reactions, researchers have also commonly explored controlled release of immunomodulatory agents (*e.g.*, nitric oxide,^31^ IL-4,^32^ IFNγ^32^) and surface functionalization with bioactive coatings (*e.g.*, IL-4,^33^ ECM components^17^). Overall, these contemporary approaches highlight the importance of tissue and immune response considerations in the design and translation of next-generation biomaterials.

Given that type I collagen represents the major structural and mechanical framework of tissues, numerous collagen-based biomaterials exist within the market today. Scaffolds fashioned from decellularized animal and human tissues represent a prominent type of collagen-based product. To create these products, tissues are processed to eliminate cellular components (*via* physical, chemical, and/or enzymatic methods) while maintaining the complex molecular composition, architecture, mechanical properties, and bioactivity inherent to the ECM.^4^ Other collagen-based products, including freeze-dried collagen sponges, are fabricated from more refined starting materials, namely tissue particulate consisting of microfibrillar collagen or collagen hydrolysates, with hydrolysates representing denatured and/or enzymatically treated collagen molecules cleaved into shorter chains of amino acids.^4,34^ All commercial collagen-based materials, regardless of source material and method of manufacture, engage innate and adaptive immune systems, resulting in a distinct FBR, commonly referenced as constructive remodeling, that features immune-mediated material bioresorption (phagocytosis and proteolytic breakdown) and fibrous tissue formation and remodeling.^35–38^ Depending on a number of factors (*e.g.*, source material, extent of decellularization, processing and exogenous crosslinking, material configuration, implant location), tissue responses can vary between collagen-based products, affecting macrophage polarization, number and distribution of FBGCs, degradation rates, and tissue response outcomes.^39^ Overall, the complexity of present-day collagen-based biomaterials and their associated design poses ongoing challenges to the elucidation of their mechanism of action and predictive modulation of their immune response.^6^

Our design strategy for next-generation engineered collagen materials employs a unique starting material consisting of a polymerizable type I collagen protein known as oligomeric collagen (Oligomer; also known as Collymer™). Oligomer is readily extracted and purified from porcine dermis, as well as other collagen-containing tissues from animal and human sources, thereby eliminating cellular and other immunogenic components.^40,41^ When acidic solutions of Oligomer are brought to physiologic conditions (*e.g.*, pH, ionic strength, and electrolyte composition) by mixing with a buffer, polymerization is initiated, giving rise to highly-interconnected and physically-stable fibrillar scaffolds, without use of exogenous additives or crosslinking agents.^40–42^ To date, engineering design tools (*e.g.*, computational modeling) and scalable fabrication methods (*e.g.*, compression densification, extrusion) have been applied for customization of wide variety of collagen polymeric material formats, including *in situ* scaffold-forming formulations, high-strength thin materials (*e.g.*, sheets, cylinders), compression-resistant constructs, and materials with gradient or aligned fibrillar microstructures.^42–50^*In vivo*, these biomaterials exhibit a unique noninflammatory, regenerative healing response with no FBR or immune-mediated bioresorption, which has been documented by implanting various material formats in a number of different anatomical locations, including dermis, subcutaneous tissue, skeletal muscle, intraperitoneal cavity, larynx, and breast.^44,46–50^

**Fig. 1:**
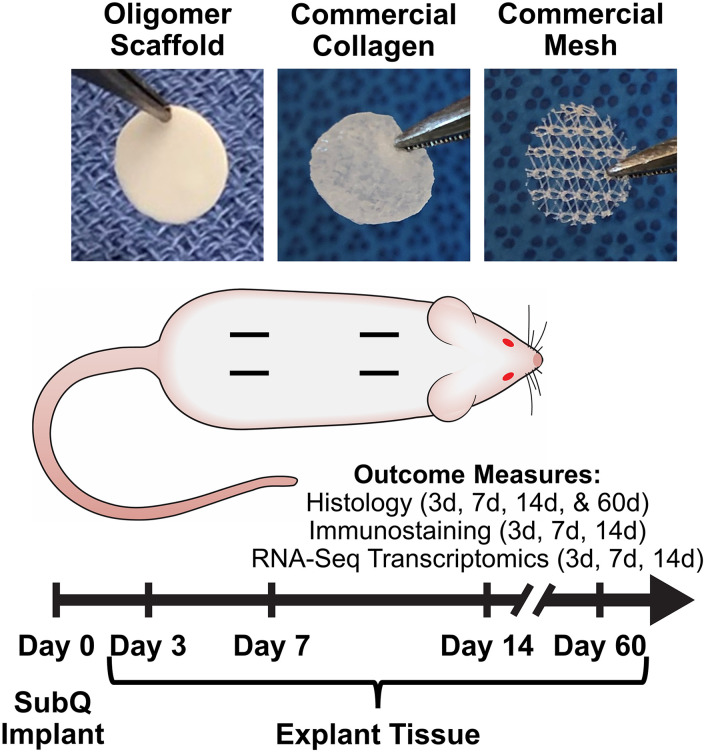
Overview of rat subcutaneous implant study design. Images show material test samples prior to implantation. Four subcutaneous pockets were created on the dorsum of each animal and randomly assigned to material and sham control groups. Measured outcomes were evaluated at 3-day, 7-day, 14-day, and 60-day time points as indicated.

To extend this work and continue to foster the bench-to-bedside translation of this biopolymer technology, we conducted a rat subcutaneous implantation study (Fig. 1) to systematically and mechanistically define the immune response and tissue reaction of a high-density Oligomer scaffold compared to commercial biomaterials with well characterized and distinguishable tissue responses, ranging from rapidly bioresorbable to nonresorbable. HeliCote® was selected as a representative bioresorbable collagen sponge material that is routinely used for wound and dental applications. HeliCote is bioengineered from bovine tendon microfibrillar collagen particulate that has been lyophilized and subjected to dehydrothermal crosslinking, yielding a material that bioresorbs within 10 to 14 days according to manufacturer specifications. Prolene® mesh, on the other hand, was selected as an exemplar nondegradable biomaterial synthesized from the thermoplastic polymer polypropylene. Because of superior burst and tensile strength, Prolene meshes are routinely used to reinforce tissues, including those associated with ventral and inguinal hernias, pelvic organ prolapses, and stress urinary incontinence. Study outcomes, which were measured at 3-, 7-, and 14-days following implantation, included gross and histological assessments of tissue reactions, immune cell identification *via* immunostaining, and RNA-sequencing (RNA-seq) transcriptomics analysis, which provided expression levels of roughly 12,000 gene products. Gross and histological assessments were also performed 60 days following Oligomer scaffold implantation to define more long-term tissue responses. Collectively, results from this study further demonstrate that engineered collagen polymeric materials fashioned from Oligomer elicit a unique regenerative mechanism of action that benefits from host immune tolerance. Additionally, results suggest new paradigms where inflammation and immune-mediated bioresorption of collagen materials are not required for desirable endogenous tissue regeneration outcomes.

## 2. Materials and methods

### 2.1 Biomaterials preparation

Custom-fabricated high-density scaffolds prepared from type I oligomeric collagen (diameter: 6.3 cm; thickness: ∼500 μm; collagen content: ∼200 mg/cm^3^) were obtained from GeniPhys, Inc. (Zionsville, IN, USA). HeliCote, a commercially available collagen wound dressing was purchased from Integra LifeSciences Corporation (Princeton, NJ, USA). Polypropylene surgical mesh (Prolene Mesh) was purchased from Ethicon (Raritan, NJ, USA). Test sample materials were created using a 10 mm biopsy punch (Robins Instruments, Chatham, NJ, USA). Oligomer scaffold and commercial collagen test samples were created under aseptic conditions; commercial mesh test samples were autoclaved prior to implantation.

### 2.2 Rat subcutaneous implantation

Material biocompatibility and immune response of Oligomer scaffold compared to commercial implant materials was assessed using a rat subcutaneous implant model as summarized in Fig. 1. All animal studies were performed according to a protocol approved by the Purdue University Institutional Animal Care and Use Committee and following AAALAC guidelines. Sprague-Dawley rats (Charles River Laboratories, Wilmington, MA, USA) weighing between 250 g and 350 g were used for the study. After induction of anesthesia, the animal's dorsum was shaved, scrubbed with surgical scrub from hip to shoulder, and allowed to dry. Four lateral incisions, approximately 1 cm in length, were made on both sides of the back, parallel to the sagittal plane. The fascia was bluntly dissected to form a small pocket just lateral to the incision. Test materials (circular, 10 mm diameter) were implanted subcutaneously just beneath the panniculus carnosus muscle, and the incision site closed with staples. For sham controls, the same procedure was applied; however, no material was implanted. The four sites within each animal were randomly assigned to the three types of material and a sham control (*n* = 8 for material and sham control groups per time point). At time points representing 3, 7, and 14 days, animals were euthanized, and the dorsum subcutaneous tissue was exposed and photographed. Tissue explants and associated biomaterials were then processed and submitted for immunostaining, histological analysis, and RNA-seq analysis. To evaluate more long-term outcomes, additional animals were implanted with Oligomer scaffold only (*n* = 8), with gross and histological tissue reaction assessments performed after 60 days. Subcutaneous tissue from normal, untreated rats was used as reference normal tissue.

### 2.3 Histological analysis and immunostaining

Processing for histological evaluation involved excision of material implant and sham sites along with a margin of surrounding normal tissue. Tissue explants were fixed in 10% neutral buffered formalin, embedded in paraffin, sectioned, and stained with hematoxylin and eosin (H&E) or Masson's Trichrome (MTC). For immunohistochemistry, sections were deparaffinized, rehydrated, processed for antigen retrieval, and stained with mouse anti-rat CD68 antibody (pan macrophage marker; 1:100; Bio-Rad, Herceles, CA, USA), rabbit anti-rat CD3 (T cell marker; 1:1000; Abcam, Waltham, MA, USA), rabbit anti-rat CD4 (T helper cell marker; 1:1200; Abcam), mouse anti-rat CD8a (cytotoxic T cell marker; 1:200; ThermoFisher Scientific, Waltham, MA, USA), rabbit anti-rat Foxp3 (Treg marker; 1:300; Abcam), or mouse anti-rat IL-17 (Th17 marker; 1:1000; Santa Cruz Biotechnology, Dallas, TX, USA). Slides were then treated with ImmPRESS® HRP Horse Anti-Mouse IgG Polymer Detection Kit (Vector Laboratories, Burlingame, CA, USA) or ImmPRESS HRP Horse Anti-Rabbit IgG Polymer Detection Kit (Vector Laboratories) and counterstained with hematoxylin. Histological assessments were performed by an independent pathologist in a blinded fashion.

### 2.4 RNA-seq analysis

Each material implant and sham site, along with a specified margin of surrounding subcutaneous tissue, was carefully dissected away from the rest of the skin layers and immediately snap-frozen in liquid nitrogen. Samples were mechanically homogenized using an Omni tissue homogenizer (Omni International, Kennesaw, GA, USA) and total RNA isolated using TRIzol (Invitrogen, Carlsbad, CA, USA). Total RNA was further purified with an RNase-Free DNase treatment for 20 minutes (Qiagen, Hilden, Germany), followed by clean-up using the RNeasy MinElute Cleanup kit (Qiagen) according to manufacturer's instructions. Samples were then submitted to the Indiana University School of Medicine Center for Medical Genomics for RNA-seq analysis. Total RNA was first evaluated for its quantity and quality using Agilent Bioanalyzer 2100 (Agilent Technologies, Santa Clara, CA, USA). Total RNA (100 ng) was used for library preparation with the KAPA mRNA Hyperprep Kit (KK8581) (Roche, Wilmington, MA, USA) following the manufacturer's protocol. Each resulting uniquely dual-indexed library was quantified and quality assessed by Qubit and Agilent Bioanalyzer, and multiple libraries were pooled in equal molarity. The pooled libraries were sequenced with 2×100 bp paired-end configuration on an Illumina NovaSeq 6000 sequencer (Illumina, San Diego, CA, USA) using the v1.5 reagent kit. The sequencing reads (25–30 million read pairs per sample) were first quality checked using FastQC (v.0.11.5, Babraham Bioinformatics, Cambridge, UK) for quality control. The sequence data were then mapped to the rat reference genome rn6 using the RNA-seq aligner STAR (v.2.5)^51^ with the following parameter: “–outSAMmapqUnique 60”. To evaluate quality of the RNA-seq data, the number of reads that fell into different annotated regions (exonic, intronic, splicing junction, intergenic, promoter, UTR, *etc*.) of the reference genome was assessed using bamutils (from ngsutils v.0.5.9).^52^ Uniquely mapped reads were used to quantify the gene level expression employing featureCounts (subread v.1.5.1)^53^ with the following parameters: “-s 2 -Q 10”. The data was normalized using TMM (trimmed mean of M values) method. Differential expression analysis was performed using edgeR (v.3.12.1).^54,55^ False discovery rate (FDR) was computed from *p*-values using the Benjamini–Hochberg procedure.

### 2.5 Statistics

Kyoto Encyclopedia of Genes and Genomes (KEGG) pathway enrichment was completed using the clusterProfiler package in R, and enriched pathways were displayed using the barplot function. For individual gene results, data was compiled using GraphPad Prism 6 (GraphPad Software, San Diego, CA, USA). Statistical significance was determined through ANOVA with Tukey *post-hoc* analysis (*p* < 0.05) using JMP Pro 15 (SAS Institute, Cary, NC, USA).

## 3. Results

### 3.1 Oligomer scaffold avoids classic FBR with minimal immune cell activation and accumulation

**Fig. 2:**
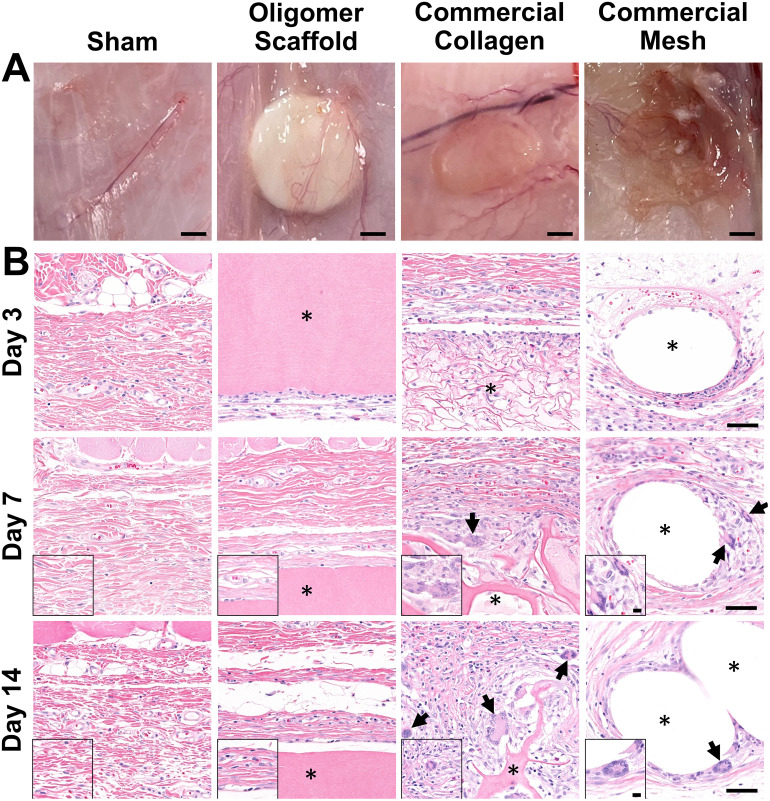
Oligomer scaffold shows high biocompatibility, with minimal immune cell accumulation and no evidence of FBGC formation. (A) Images of excised materials and sham site with surrounding tissue margin at 14-day time point. Scale bars: 2 mm. (B) Cross-sections stained with H&E of sham surgical site and explanted materials with surrounding tissue margin at 3-day, 7-day, and 14-day time points. Asterisk (*) denotes material implants and arrows indicate FBGCs. Scale bars: 50 μm; inset: 10 μm.

The well-established rat subcutaneous model^56,57^ was employed to characterize and compare the local immune and tissue response of Oligomer scaffolds with those for conventional biological and synthetic materials. All animals remained healthy and displayed expected weight gains throughout the study. Qualitative assessment of surgical sites showed no evidence of erythema (redness); however, roughly 50% of commercial mesh sites across all time points showed moderate edema (swelling) and seroma formation. Based on gross and histological assessments of material implant and sham sites, it was apparent that Oligomer scaffolds were highly biocompatible and elicited a distinct short-term (≤14 days) tissue response compared to the commercial materials. To confirm that Oligomer scaffolds did not elicit any long-term deleterious reactions, additional 60-day gross and histological assessments were performed only for this material. Grossly, sham and Oligomer sites showed no obvious tissue reaction or evidence of fibrous tissue overgrowth at both 14-day (Fig. 2A) and 60-day (Fig. S1A†) time points. Additionally, Oligomer scaffolds maintained their white appearance and material volume. Consistent with previous reports,^49^ macrovascularization was evident along the Oligomer scaffold exterior (Fig. 2B and Fig. S1B, C†). By contrast, the bioresorbable commercial collagen took on a yellow coloration and decreased in volume over the 14-day study period (Fig. 2A). The mesh, as expected, elicited an obvious tissue reaction, which was evident as early as day 3. By day 14, ample fibrous connective tissue was seen surrounding and integrated within the material openings (Fig. 2A).

A detailed histological analysis of H&E- (Fig. 2B and Fig. S1B, C†) and MTC- (Fig. S2†) stained sections provided additional insights into the immunophenotypic response of Oligomer scaffolds compared to conventional implantable materials. Histologically, the Oligomer scaffold appeared as a homogenous and bright pink (eosinophilic) material that stained similarly to subcutaneous collagen (Fig. 2B and Fig. S1B, C†). At day 3, a relatively small number of mononuclear leukocytes and fibroblasts (1–2 cells thick) were seen accumulating around the Oligomer scaffold surface; however, by day 7, this cell density declined to match densities and distributions observed within the sham surgical site (Fig. 2B). Importantly, there were no FBGCs observed at any time point, demonstrating no evidence of chronic inflammation. Additionally, cell infiltration of the Oligomer scaffold was minimal, and no scaffold degradation or abnormal histopathology of the surrounding subcutaneous tissue was noted. Similar findings were observed for 60-day Oligomer scaffold implants, with no evidence of a long-term, deleterious tissue reaction grossly or histologically (Fig. S1†).

By contrast, commercial collagen and mesh showed an increase in cellularity over time, with significant infiltration around and within the materials (Fig. 2B and Fig. S2†). At 3 days, the commercial collagen appeared as an integrated meshwork of variably-sized, pink-staining particulate, which was well circumscribed and encapsulated with a robust mixture of inflammatory cells, fibroblasts, and collagen. Gradual resorption of the commercial collagen over the 14-day study period was apparent, which was accompanied by an increase in fibrous connective tissue deposition (Fig. 2B and Fig. S2†). Histological analysis also revealed progressive cell infiltration and active proteolytic degradation and phagocytosis by macrophages and FBGCs (Fig. 2B, arrows and Fig. S2†). Additionally, a moderate inflammatory reaction, characterized by infiltrating lymphocytes, plasma cells, and macrophages, was observed within the nearby subcutaneous tissue. An inflammatory-mediated FBR was also observed with the mesh. Histologically, the mesh appeared as multiple unstained spaces, which became surrounded by a mix of inflammatory cells over time and increasing amounts of fibrous connective tissue (Fig. 2B and Fig. S2†). Circumscribing the implant was a mix of neutrophils, macrophages, lymphocytes, plasma cells, and abundant plump fibroblasts. By 7 days post implantation, FBGCs were evident, with increasing numbers noted at day 14 (Fig. 2B, arrows). At all time points, a significant inflammatory reaction was noted adjacent to the mesh. This reaction was seen extending into the overlying panniculus carnosus muscle on day 14. Additionally, by 14 days, a dense capsule of fibrous connective tissue surrounded the mesh implant, indicative of a classic FBR (Fig. 2A and Fig. S2†).

**Fig. 3:**
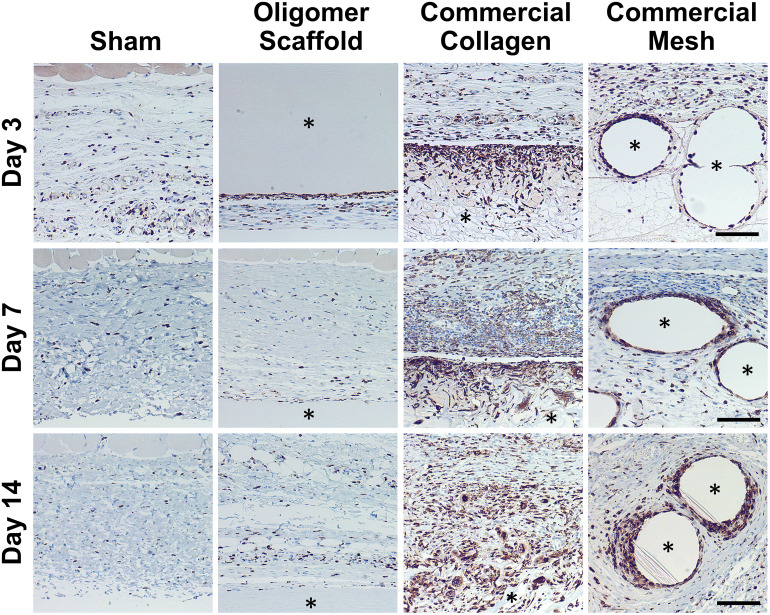
Oligomer scaffold shows modest and transient accumulation of CD68+ macrophages similar to sham control. Cross-sections immunostained (brown) for CD68 at 3-day, 7-day, and 14-day time points; counterstained with hematoxylin (blue). Asterisk (*) denotes material implants. Scale bars: 100 μm.

Because macrophages are considered major players in the FBR to biomaterials,^32,33^ immunostaining for the pan-macrophage surface marker CD68 (Fig. 3) was performed to support further spatial and temporal cellular characterization. As shown in Fig. 3, modest increases in CD68+ cells were noted within sham and Oligomer sites at day 3, with levels decreasing to normal subcutaneous levels by 14 days. On the other hand, increased numbers of CD68+ macrophages were found associated with commercial collagen and mesh implants, with the apparent density and depth of material infiltration increasing over the 14-day period (Fig. 3). For commercial collagen, high numbers of CD68+ macrophages were seen accumulating at the material-tissue interface at day 3, with significant but somewhat lesser numbers distributed throughout the adjacent subcutaneous tissue and penetrating into the material. CD68+ staining remained high at days 7 and 14 but showed more uniform distribution, as macrophages progressively infiltrated the material over time (Fig. 3). On the other hand, CD68+ macrophages formed a thin layer along the mesh surface on day 3, with even greater numbers distributed within the surrounding stroma (Fig. 3). These macrophages appeared to increase in density over the duration of the study. Overall, gross, histological, and immunostaining results suggested that the three materials elicited strikingly different tissue reactions and cellular responses, with the Oligomer scaffold showing an uncommon tissue response that was not driven by macrophages and showed no significant foreign body response or bioresorption.

### 3.2 Transcriptomics reveals molecular-level signaling profile consistent with immune tolerance for Oligomer scaffold

To further define differential immune responses at the molecular level, RNA-seq gene expression analyses were performed for material and sham groups, using normal, untreated subcutaneous tissue as a reference. Prior to RNA isolation, material implant and sham surgical sites, along with roughly a 2 mm tissue margin, were dissected away and excised from the surrounding skin layers. It is notable that isolated total RNA quantities varied between groups, likely reflecting differences in explant cellularity. More specifically across all time points, highest total RNA yields were obtained for mesh, followed by commercial collagen, Oligomer scaffold, and sham.

**Fig. 4:**
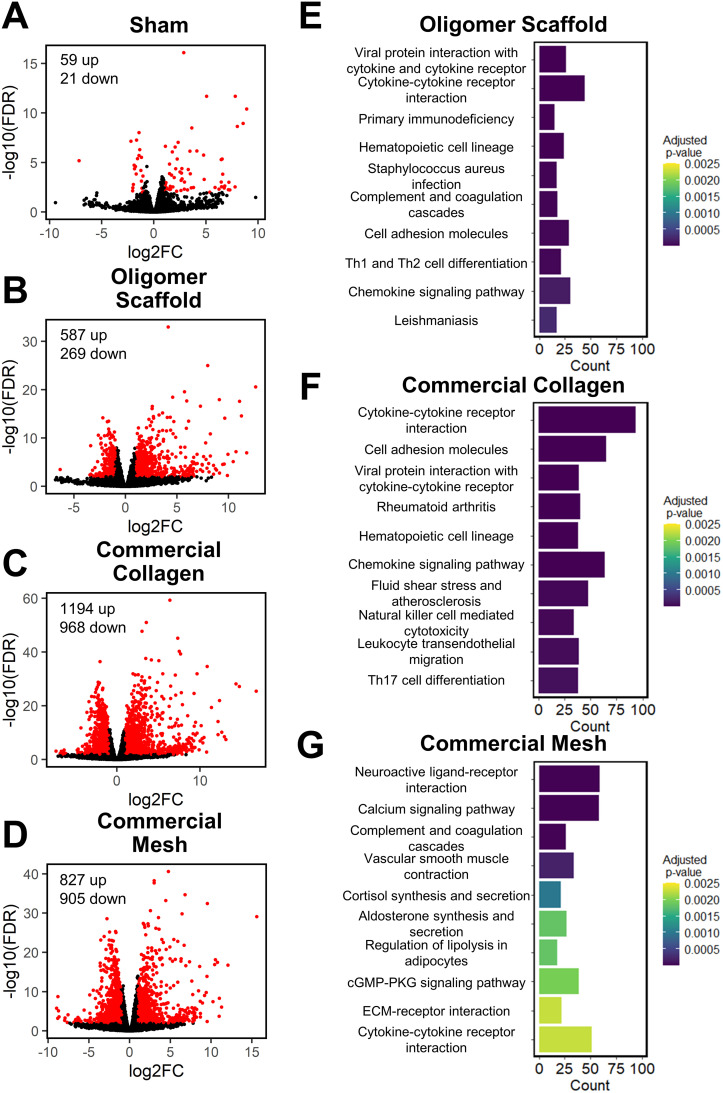
Volcano and KEGG analyses at 14-day time point suggest Oligomer has a different molecular-level tissue response pathway compared to conventional implant materials. (A–D) Volcano plots of (A) sham, (B) Oligomer scaffold, (C) commercial collagen and (D) commercial mesh, featuring differentially expressed genes with |log_2_FC| > 1 & FDR < 0.01 (red) compared to normal tissue. (E–G) KEGG pathway enrichment analysis of the 10 most statistically significant pathways for (E) Oligomer scaffold, (F) commercial collagen, and (G) commercial mesh compared to normal tissue, featuring the number of DEGs (counts) occurring within each pathway; *n* = 5–6 per group.

Principal component analysis (PCA) at each time point revealed different group profiles, with distinct clusters converging by day 14 (Fig. S3†). Given that day 14 yielded the most stable, distinct responses, this time point was the primary focus for further volcano plot and KEGG analyses. Volcano plots, which visualize differentially expressed genes (DEGs), revealed little difference between sham and normal tissue at day 14, with only 80 differentially expressed genes (DEGs) identified under the condition of |log_2_ fold changes| > 1 and FDR < 0.01 (Fig. 4A). For Oligomer scaffold, 856 DEGs were identified, which was substantially less than commercial collagen (2162 DEGs) and mesh (1732 DEGs) (Fig. 4B–D). KEGG overenrichment analyses further informed the transcriptomic analysis through quantification of the pathways most significantly associated with DEGs for each material, and the statistical relevance of the relation to that pathway was also evaluated (adjusted *p*-values <0.05). As shown in Fig. 4E–G, all three materials showed engagement of several immune-related pathways, such as cytokine–cytokine receptor interaction, hematopoietic cell lineage, cell adhesion molecules, complement and coagulation cascades, and chemokine signaling pathways. However, the number of DEGs within these pathways was lowest for Oligomer scaffold. Interestingly, KEGG analysis also suggested differential involvement of T cell mediated adaptive immune pathways for Oligomer scaffold and commercial collagen, while the mesh showed primarily innate immunity pathway activation (Fig. 4E–G). Mesh implantation also led to overenrichment of the ECM-receptor pathway, which is consistent with fibrosis (Fig. 4G).^58^

**Fig. 5:**
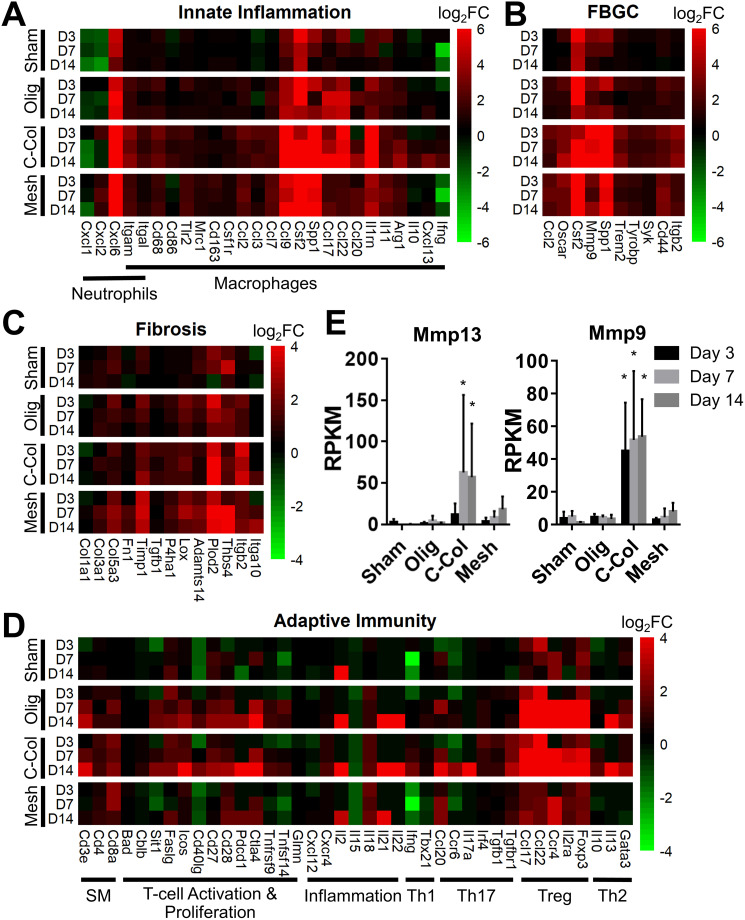
Oligomer scaffold displays distinct molecular signature suggestive of immune tolerance. Gene subsets for (A) innate inflammation, (B) FBGC, (C) fibrosis, and (D) adaptive immunity as determined by RNA-seq for sham, Oligomer scaffold (Olig), commercial collagen (C-Col) and commercial mesh (Mesh) at specified time points; normalized to normal tissue; *n* = 5–6 per group. (E) Reads per kilobase of transcript per million reads mapped (RPKM) values for select genes associated with collagen resorption (mean ± SD). Asterisk (*) denotes statistical difference compared to sham; *p* < 0.05; *n* = 5–6 per group. SM: surface markers.

Informed by KEGG analysis outcomes, more detailed evaluations of gene subsets were conducted, beginning with those related to innate immune cell (*e.g.*, neutrophil and macrophage) phenotype and activation. As shown in Fig. 5A, the profiles for Oligomer scaffold and sham were most similar and paralleled histological observations. Both showed transient upregulation of several innate inflammation genes, including the macrophage associated genes *Ccl9*, *Csf2*, *Spp1*, and *Il1rn*, with levels decreasing over the 14-day study period. A notable exception was *Ccl17* and *Ccl22*, which for Oligomer scaffold maintained increased expression at 7 and 14 days (FDR < 0.01; *Ccl17*: log_2_FC = 5.3 at 7 days, log_2_FC = 4.5 at 14 days; *Ccl22*: log_2_FC = 5.4 at 7 days, log_2_FC = 4.9 at 14 days). Interestingly, these genes encode chemokines known to recruit Tregs.^7^ Additionally, high and sustained expression of *Cxcl6*, a chemoattractant of neutrophils and potent angiogenic chemokine,^59^ was observed for Oligomer scaffold but not sham (Fig. 5A). By contrast, commercial collagen and mesh showed broad upregulation of innate inflammation genes with no discernible temporal trends (Fig. 5A). At 14 days, commercial collagen showed high expression of genes associated with neutrophil chemotaxis, angiogenesis, and macrophage lineage, activation, and chemotaxis (FDR < 0.01: *Cxcl6* log_2_FC = 10.0, *Itgam* log_2_FC = 3.4, *Cd68* log_2_FC = 2.7, *Cd86* log_2_FC = 1.7, *Ccl2* log_2_FC = 1.5, *Ccl3* log_2_FC = 2.2, *Ccl7* log_2_FC = 2.0, *Ccl9* log_2_FC = 7.5, *Csf2* log_2_FC = 6.6, *Spp1* = 9.7). Upregulation of macrophage phenotype and function genes was also observed with commercial collagen, including *Ccl17*, *Ccl22*, *Ccl20*, *Il1rn*, *Arg1*, *Il10*, *Cxcl13*, and *Ifng*, many of which foster engagement of the adaptive immune system.^18,60^ The heatmaps for mesh and commercial collagen were most similar with the exceptions that *Itgam*, *Cd86*, *Ccl3*, *Ccl17*, *Ccl22*, *Ccl20*, *Il1rn*, *Arg1*, and *Cxcl1* showed somewhat lesser or no upregulation, and *Ifng* was downregulated for the mesh group (Fig. 5A).

Since FBGCs, which are a hallmark of FBRs, appeared to play a significant role in the tissue responses to commercial collagen and mesh but not Oligomer scaffold, signaling pathways associated with macrophage fusion and FBGC formation were also evaluated in detail. As shown in Fig. 5B, FBGC heatmaps for Oligomer scaffold and sham appeared largely similar, suggesting a transient response to the surgical procedure and confirming a lack of FBGC involvement. Commercial collagen, on the other hand, showed upregulation of all genes within the FBGC subset. In fact, factors known to stimulate macrophage fusion, namely *Oscar* (log_2_FC = 4.4 at 14 days), *Csf2* (log_2_FC = 6.6 at 14 days), *Mmp9* (log_2_FC = 6.4 at 14 days), and *Spp1* (log_2_FC = 9.7 at 14 days), were amongst the most highly upregulated factors throughout the 14-day study period (Fig. 5B).^13,58,61^ Fusion process genes (*Trem2*, *Tyrobrp*, and *Syk*) and markers associated with FBGC and phagosomes (*Cd44* and *Itgb2*) also showed moderate upregulation across all time points.^10,13^ Again, mesh exhibited a similar profile to commercial collagen, with the exceptions that lesser upregulation of *Mmp9* (log_2_FC = 3.7 at 14 days) and *Itgb2* (log_2_FC = 2.2 at 14 days) and no upregulation of *Syk* were observed. These differences are likely associated with divergent pathways and outcomes for FBRs, with commercial collagen and mesh yielding bioresorption and fibrotic encapsulation, respectively.

Additional mechanistic insight into the differential material responses was gleaned by analyzing gene subsets associated with ECM synthesis, assembly, and degradation. Once again, Oligomer scaffold and sham profiles were largely similar, with various genes showing modest and transient upregulation, likely owing to the surgical procedure (Fig. 5C). Transcriptomics showed that Oligomer scaffold, like sham, maintained baseline expression of matrix metalloproteinases, *Mmp13* and *Mmp9*, and the tissue inhibitor of matrix metalloproteinases (*Timp1*), further confirming the lack of observed bioresorption, material degradation, and fibrotic response (Fig. 5E). As shown in Fig. 5C and 5E, only commercial collagen featured a robust and consistent upregulation of *Mmp9* (log_2_FC = 6.5 at 14 days), *Mmp13* (log_2_FC = 12.6 at 14 days), and *Itgb2* (log_2_FC = 3.5 at 14 days). Upregulation of this gene combination corroborated the active phagocytosis and rapid ECM proteolysis observed histologically.^13,58,62^ Modest upregulation of genes favoring ECM deposition and maintenance was also observed, including *Tgfb1* (log_2_FC = 1.1 at 14 days), *P4ha1* (log_2_FC = 1.3 at 14 days), *Plod2* (log_2_FC = 4.9 at 14 days), and *Timp1* (log_2_FC = 2.6 at 14 days) (Fig. 5C). On the other hand, the mesh showed engagement of pathways consistent with fibrosis and fibrotic capsule formation,^63,64^ with strong upregulation of *Plod2* (log_2_FC = 3.9 at 14 days), *Thbs4* (log_2_FC = 5.0 at 14 days) as well as *Timp1* (log_2_FC = 3.2 at 14 days) (Fig. 5C). This was complemented by more moderate upregulation of additional ECM synthesis and assembly genes,^65^ including *Col3a1* (log_2_FC = 1.9 at 14 days), *Col5a3* (log_2_FC = 2.3 at 14 days), *Fn1* (log_2_FC = 1.4 at 14 days), *P4ha1* (log_2_FC = 1.1 at 14 days), *Adamts14* (log_2_FC = 2.0 at 14 days), and *Lox* (log_2_FC = 1.8 at 14 days) (Fig. 5C).

Given the known crosstalk between innate and adaptive immune components and the KEGG analyses implicating certain T lymphocyte pathways, a more in-depth analysis of adaptive immunity genes was performed. As shown in Fig. 5D, the groups showed distinct profiles, with Oligomer scaffold and commercial collagen exhibiting the most similarities. Both groups showed upregulation of a broad array of genes associated with T cell surface markers (*CD3e*), T cell activation and proliferation (*Sit1*, *Icos*, *Cd27*, *Cd28*, *Pdcd1*, *Ctla4*), and inflammation (*Il18*, *Il21*), especially at later time points (Fig. 5D). Additionally, both Oligomer and commercial collagen exhibited similar expression patterns for genes associated with Treg (*Foxp3*, *Il2ra*, *Ccr4*) and Th2 (*Gata3*, *Il13*) pathways (**Olig**: *Foxp3* log_2_FC = 5.3, *Il2ra* log_2_FC = 4.7, *Ccr4* log_2_FC = 6.2, *Gata3* log_2_FC = 2.3, *Il13* log_2_FC = 5.8 at 14 days; **C-Col**: *Foxp3* log_2_FC = 6.6, *Il2ra* log_2_FC = 5.8, *Ccr4* log_2_FC = 7.4, *Gata3* log_2_FC = 3.0, *Il13* log_2_FC = 7.1 at 14 days). Interestingly, heatmaps also revealed a number Th1/Th17 pathway genes that were only upregulated for commercial collagen (Fig. 5D). These factors included the characteristic cytokine of Th1 and Th17 cells,^21^*Il17a* (log_2_FC = 8.3 at 14 days); *Irf4* (log_2_FC = 1.1 at 14 days), a transcription factor implicated in induction, amplification, and stabilization of the Th17 phenotype;^66^*Ccr6* (log_2_FC = 2.5 at 14 days), a receptor upregulated on Th17 cells; *Ccl20* (log_2_FC = 3.8 at 14 days), a known Th17 and Treg chemokine;^67^ and *Ifng* (log_2_FC = 2.0 at 14 days) and *Tgfb1* (log_2_FC = 1.1 at 14 days). The heatmap for mesh (Fig. 5D), on the other hand, suggested only modest T cell engagement, which is consistent with previous reports for nonabsorbable materials.^63,68,69^ Similarly, sham implantation showed minimal engagement of the adaptive immune system (Fig. 5D). Collectively, transcriptomics data corroborated gross and histological findings and further documented that Oligomer-based materials have an uncommon tissue response and mode of action, where it engages cells to yield immune tolerance.

**Fig. 6:**
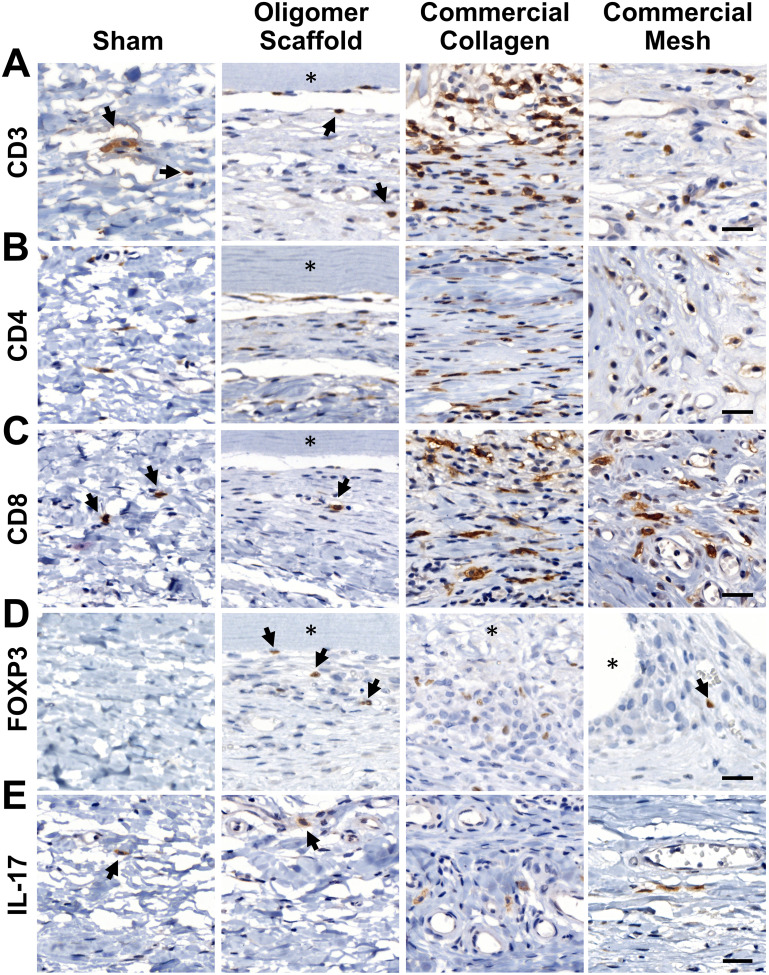
Oligomer scaffold shows a subtle T cell response involving Foxp3+ Tregs at 14-day time point. Cross-sections immunostained (brown) for (A) CD3, (B) CD4, (C) CD8, (D) Foxp3, and (E) IL-17; counterstained with hematoxylin (blue). Asterisk (*) denotes material implants and arrows highlight examples of positively stained cells. Scale bars: 25 μm.

Given that transcriptomics data suggested a differentiating adaptive immune response for Oligomer scaffold, immunostaining for T cell markers CD3, CD4, CD8, Foxp3, and IL-17 was conducted to corroborate gene expression profiles. As shown in Fig. 6A, the 14-day tissue reaction associated with commercial collagen comprised the greatest number of CD3+ lymphocytes, with more moderate numbers identified around mesh and only a few associated with Oligomer scaffold and sham. Further analysis of subtypes showed that commercial collagen featured a mixed population of CD4+ T helper and CD8+ cytotoxic T cells (Fig. 6B and C), including Foxp3+ and IL-17+ T helper cell subtypes (Fig. 6D and E). Mesh also showed a mix of CD4+ and CD8+ cells (Fig. 6B and C), with lesser IL-17+ (Fig. 6E) and few Foxp3+ (Fig. 6D) cells. Of the limited lymphocytes associated with Oligomer scaffolds and sham, Oligomer showed a modest bias towards increased Foxp3+ Treg cells (Fig. 6). Overall, immunostaining results corroborated transcriptomic findings, confirming that Oligomer scaffold favored a subtle T cell response involving Tregs while commercial collagen and mesh induced robust and mixed adaptive immune cell engagement.

## 4. Discussion

**Fig. 7:**
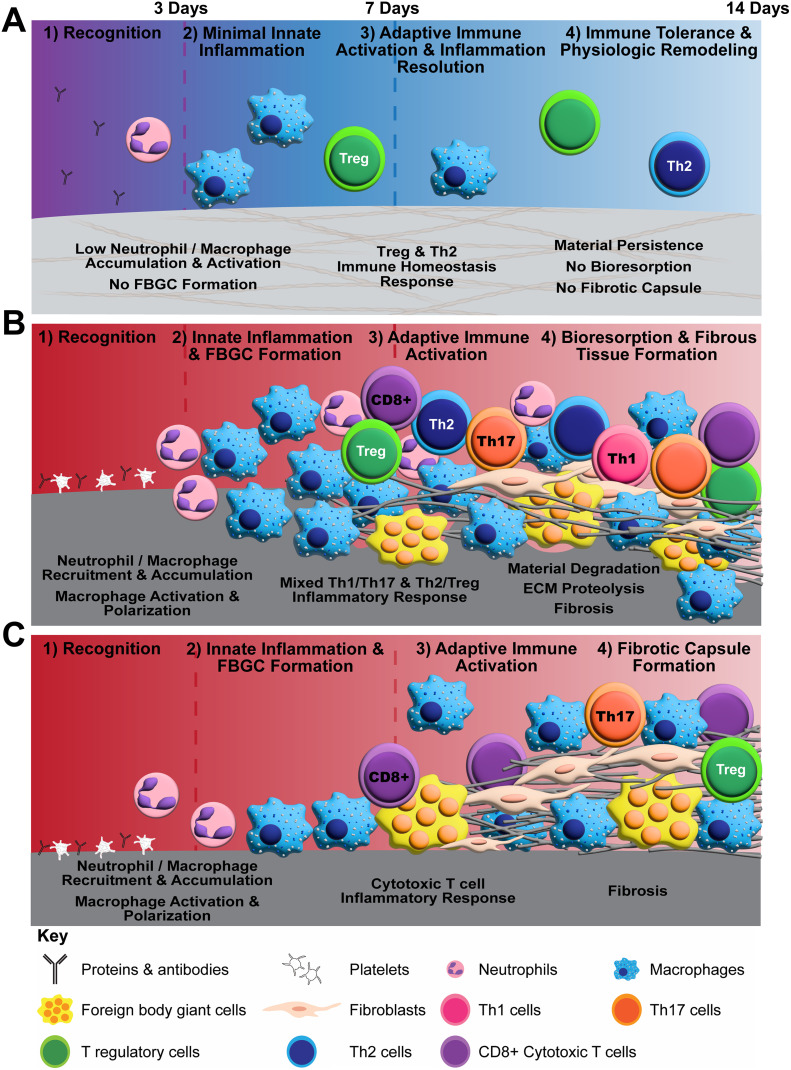
Schematics summarizing proposed differential biomaterial implant responses observed in rat subcutaneous implant model. (A) Proposed mechanism of action for Oligomer scaffold featuring immune tolerance with minimal innate immune activation, no FBGC formation, no resorption, and no fibrosis. (B) Conventional collagen biomaterial response featuring activation of innate and adaptive pathways, FBGC formation, resorption, and fibrosis. (C) Conventional synthetic implant response featuring activation of innate inflammation, FBGC formation, and fibrotic capsule formation.

Polymerizable oligomeric collagen supports the engineering design and custom fabrication of a broad variety of collagen polymeric material formats that have the potential to improve device-tissue interfaces as well as address today's unmet tissue reconstruction, restoration, and regeneration needs. Studies conducted to date suggest that Oligomer materials avoid inflammation and favor regenerative mechanochemical signaling with restoration of site-appropriate tissue histology and function that, to the best of our knowledge, has not been observed previously with other synthetic, natural, or nature-derived biomaterials.^44,47,50^ In the present study, gross, histological and transcriptomic assessments of local tissue reactions following subcutaneous implantation allowed further elucidation and differentiation of the Oligomer material immune response compared to conventional implantable materials. Oligomer scaffold showed a unique tissue response, which we term regenerative remodeling, that featured a transient, low-level innate immune reaction, similar to sham surgical control, and modest adaptive immune cell presence (Fig. 7A). The high-density scaffold maintained its structural and physical features, with no evidence of immune-mediated bioresorption. An interesting finding was that Th2 and Treg cell markers and signaling pathways were upregulated, implicating these cells in the host's perception of Oligomer scaffolds as “self” rather than “foreign”. Consistent with conventional collagen-based materials,^36,38,70^ the commercial collagen displayed a constructive remodeling phenotype, featuring large numbers of macrophages and FBGCs, a mixed Th1/Th17/Th2/Treg lymphocytic reaction, material resorption *via* phagocytosis and proteolysis, and fibrous tissue formation and remodeling facilitated by fibroblasts (Fig. 7B). The non-degradable commercial mesh, on the other hand, showed a robust innate immune response, a moderate lymphocytic reaction, and fibrotic encapsulation (Fig. 7C).

Neutrophils and macrophages are dominate innate immune cell players during both early and late phases of FBRs to implantable materials, including metals (*e.g.*, titanium), synthetic polymers (*e.g.*, silicone, polypropylene, polycaprolactone), biological materials (*e.g.*, collagen sponges, decellularized ECM scaffolds), and nature-derived materials (*e.g.*, alginate, silk).^17,19,22,24,68^ Targeted cell knockout or depletion studies show that macrophages are primary contributors to the fibrotic response,^69^ and both neutrophils and macrophages participate in degradation of collagen-based materials.^71^ Consistent with this notion as well as other published preclinical and human clinical studies,^72–77^ we show that the commercial mesh, when implanted subcutaneously, induced fibrotic capsule formation driven by large numbers of macrophages and FBGCs. The filamentous mesh structure supported rapid cell accumulation around and throughout the mesh as well as an extended inflammatory reaction that progressed outward into the surrounding normal tissue over time. Commercial collagen featured a similar temporal accumulation of macrophages and FBGCs; however, its material composition and porous microstructure led to a different cellular infiltration pattern. In this case, cells penetrated deeper into the material from the tissue interface over time, with progressive material degradation. A distinct response was observed with Oligomer scaffold, where macrophage activation appeared transient, similar to the sham control. Because macrophages did not appear to drive the Oligomer scaffold response, definition of macrophage polarization according to traditional M1/M2 types was considered outside the scope of this work. Broadl*y*, transcriptomics showed common and persistent upregulation of *Cxcl6*, *Cd68*, *Ccl9*, *Csf2*, and *Spp1* for both commercial materials, and increased expression of *Cd86*, *Ccl2*, *Il1rn*, *Arg1*, *Ccl20*, *Ccl22*, *Il10*, *Cxcl13*, and *Ifng* for commercial collagen. Such inflammatory cytokine and cell marker profiles are consistent with mixed or hybrid macrophage phenotypes as suggested by other published work.^78,79^ For both materials, FBGCs were readily identifiable at 7- and 14-day time points, with corroborating upregulation of macrophage fusion genes (*Oscar*, *Csf2*, *Spp1*, *Trem2*, and *Tyrobp*) and FBGC surface markers (*Cd44*). *Mmp9*, *Mmp13*, and *Itgb2* expression was enhanced only for commercial collagen, which is consistent with the active phagocytosis and proteolysis of collagen materials.^58,62^

By contrast, Oligomer scaffold showed minimal innate immune cell accumulation and activation, with associated histological findings and heatmaps that largely mirrored the extent and temporal response of the sham surgical group. A notable exception was the early and persistent upregulation of *Ccl22* and *Ccl17*, which was observed with Oligomer scaffold but not sham. These macrophage and dendritic cell derived chemokines are potent recruiters of Th2 and Treg cells, which are required for maintenance of self-tolerance, controlling inflammation, and preventing autoimmune diseases.^80–83^ It is reasonable to suppose that the apparent recognition of Oligomer scaffold as “self” by the host innate immune system is owing to its composition and structural features. Immune reactions elicited by conventional collagen materials, whether fabricated from decellularized tissues or processed tissue particulate (*e.g.*, microfibrillar collagen, hydrolysates, gelatin) are largely driven by immunogenic cell remnants (*e.g.*, membrane and intracellular), non-collagenous molecules, as well as leachables (*e.g.*, detergents) and deleterious effects caused by material processing and formatting (*e.g.*, denaturation, exogenous crosslinking, sterilization).^39,84^ By contrast, Oligomer materials are fabricated from highly-purified type I collagen protein building blocks, comprising full-length collagen molecules held together by the natural, non-reducible intermolecular crosslink chemistry known as histidinohydroxylysinonorleucine (HHL).^41,85^ The component collagen polypeptide chains (*e.g.*, α1 and α2) and intermolecular crosslink chemistry are highly conserved across species,^86^ with reported values for porcine and human sequence homologies being 97% and 94% for component α1 and α2 chains, respectively.^87^ Relevant to the present study, sequence homology between porcine and rat collagen is slightly less but still high, with values of 91% for the α1 chain and 90% for α2 chain. Further, the structure of Oligomer scaffolds closely resembles native collagen found within tissue ECMs,^42^ which likely contributes to its noninflammatory response. The molecular self-assembly (polymerization) of Oligomer occurs at physiologically-relevant pH and ionic strength and is driven by hydrophobic and electrostatic interactions, yielding highly interconnected fibrillar collagen materials.^41^ The component D-banded fibrils, with roughly 67 nm periodicity, are similar to those found in connective tissues,^42^ facilitating cellular recognition, macromolecular interactions, and mechanochemical signaling. In summary, it is reasonable to suppose that tissue response differences between Oligomer scaffold and commercial collagen are largely driven by differences in both molecular composition and multi-scale structural features.

While the innate immune system plays a critical role in biomaterial FBRs, more recent investigations have focused on adaptive immune system participation (see Adusei *et al.*, 2021 ^21^ for recent review). In short, stimulation of lymphocyte migration, proliferation, and downstream maturation occurs following innate immunity engagement through antigen-presenting dendritic cells and macrophages, along with secreted cytokines. Integrative signaling between innate and adaptive immune components, in turn, drives biomaterial responses, ranging from tissue regeneration to tissue repair (fibrosis) to chronic inflammation.^2,16^ In the present study, transcriptomics data indicated adaptive immune system engagement within the first week following Oligomer scaffold implantation, with upregulation of Th2 and Treg pathway genes dominating by 14 days. Specifically, Oligomer induced high levels of *Ccr4*, which encodes a Treg receptor that binds the recruitment chemokines CCL17 and CCL22.^83^ Treg participation was further confirmed by the upregulation of Forkhead box P3 (*Foxp3*), a transcription factor essential for Treg maturation and function, as well as Treg surface receptors *Ctla4* (cytotoxic T lymphocyte antigen), *Icos* (inducible T cell costimulatory), and *Il2ra* (interleukin 2 receptor α-chain).^80,88,89^ Previous studies have documented that recruited and resident Treg populations control inflammation after tissue injury and restrain immune responses to self and foreign antigens by modulating neutrophil and macrophage function and suppressing CD4+ and CD8+ T cell-mediated inflammation.^90,91^ Moreover, it has been suggested that Tregs directly facilitate tissue regeneration *via* activating local stem and progenitor cell populations.^92–94^ In addition to promoting Treg activity, Oligomer scaffold implantation led to increased Th2 pathway gene expression, including the key transcription factor GATA3 and cytokine IL-13.^95^ Th2 cells, in addition to Tregs, are known suppressors of Th1- and Th17-driven inflammation, with many studies suggesting a critical role of IL-13 and IL-4 activated macrophages in the resolution of inflammation and the restoration of immune homeostasis.^96,97^ Th2 cells have also been identified as a required cell type for the constructive remodeling response observed with decellularized ECM scaffolds.^78,98,99^ Overall, the promotion of Th2/Treg signaling by Oligomer scaffolds suggests that adaptive immune engagement is likely a key attribute of its unique noninflammatory mechanism of action.

Th2/Treg pathways were also upregulated by commercial collagen; however, this activity was counterbalanced by engagement of pro-inflammatory Th1 and pro-fibrotic Th17 pathways. Specifically, *Il17a*, *Ccl20*, *Irf4*, and *Ccr6*, which are Th17 pathway gene products, as well as *Ifng*, the signature gene for the Th1 pathway, were upregulated exclusively with commercial collagen, especially at later time points. Given the high numbers of macrophages with mixed phenotypical expression, the observed mixed T cell population is not unexpected. Still, the balance between Th1/Th17 and Th2/Treg pathways is an important determinant of fibrosis, as demonstrated by biomaterial outcomes and disease pathogenesis.^21,80^ Interestingly, Th17 and Treg subtypes have interconnected differentiation pathways and antagonist functions.^100,101^ Th17 cells are associated with autoimmunity, inflammation, and fibrosis whereas Tregs inhibit these processes and maintain immune homeostasis. More specifically, the extent of capsular fibrosis observed clinically for failed silicone breast and polypropylene mesh implants was shown to be dependent on a shift in the balance towards Th1/Th17 activity and inversely proportional to the number of Tregs.^74,102^ Additionally, IL-13 has been identified as a driver of myofibroblast differentiation and collagen deposition,^95,103,104^ further supporting the significance of the T helper cell balance during tissue repair. In a distinct mechanism from the commercial collagen, the lymphocytes observed histologically surrounding the commercial mesh showed CD4+ and CD8+ T cell populations, with more moderate upregulation of lymphoid-specific pathways evident from transcriptomics data across the 14-day study period. For mesh, there appeared to be greater macrophage and FBGC accumulation compared to T cell numbers that was observed histologically. In support, such results are consistent with previous studies that show that monocyte-derived macrophages dominate the chronic inflammation and FBR to polypropylene mesh at both early and late stages following implantation in mice and humans.^72^ Overall, findings from the present study further demonstrate the importance of the Th2/Treg and Th1/Th17 balance in determining material responses, with Oligomer materials exhibiting prominent Th2/Treg signaling and no evidence of Th1/Th17 activation, thereby shifting the balance towards immune tolerance and away from inflammation, material bioresorption, and fibrosis.

Present-day collagen materials, regardless of their method of fabrication, undergo immune-mediated bioresorption with fibrous connective tissue formation and remodeling.^34,105^ To control degradation rate and enhance mechanical stability, materials are routinely subjected to various types and levels of exogenous crosslinking, which increases covalent bonding between chemical moieties. Common methods employed both commercially and non-commercially include those involving glutaraldehyde, isocyanates, dehydrothermal, and carbodiimide.^4^ Not only is the specificity of these crosslinking reactions difficult to control within complex biological materials, but the extent of crosslinking is also positively correlated with enhanced FBR, fibrous capsule formation, cytotoxicity, and calcification (see Delgado *et al.*, 2015 ^39^ for review). In the present study, commercial collagen showed rapid bioresorption, which is consistent with modest crosslinking and the 10- to 14-day resorption rating of this product. As with other collagen materials, cell-mediated material phagocytosis and proteolysis was facilitated by neutrophils, macrophages, and FBGCs, as well as associated matrix metalloproteinases MMP-13 and MMP-9, which are known to degrade intact collagen fibrils and collagen fragments, respectively.^10,105,106^ This degradation process is accompanied by the influx of fibroblasts and other stromal cell populations that deposit ECM components, including collagen, and progressively remodel the fibrous connective tissue. This well-known collagen biomaterial response is referred to as “constructive remodeling”, with tissue response outcomes “culminating in the formation of a tissue that is usually site appropriate, at least partially functional, and devoid of any persistent or chronic inflammatory reaction”.^71,107^ This remodeling process does not represent tissue morphogenesis or regeneration in the true sense, but rather is best described as a departure from the default wound contraction and scar tissue formation observed with tissue injury.^34,107^ Interestingly, in most situations when conventional collagen materials are exogenously crosslinked, improved persistence is achieved, but chronic inflammation and a more robust FBR are also observed.^39^ There is an increased number and prolonged presence of macrophages and FBGCs, enhanced type 1 immune response, and enhanced fibrosis and/or fibrotic encapsulation, ultimately yielding undesirable outcomes.^39,84,107^ This has led to suppositions within the biomaterials field that inflammation and biological scaffold degradation are requisite processes to achieve favorable constructive remodeling outcomes.^107,108^

The immune response and regenerative remodeling observed following Oligomer material implantation is distinct from conventional collagen materials, thereby challenging current paradigms. Unlike commercial collagen scaffolds, Oligomer scaffolds do not undergo degradation (bioresorption) following implantation in the body. Oligomer scaffolds undergo cellular remodeling *via* mechanisms that largely involve cell-collagen mechanobiological signaling and collagen metabolism (turnover). It is notable that these mechanisms occur in absence of a material-induced inflammatory response, which is important since inflammatory signals and processes may modulate or even override these normal tissue homeostatic mechanisms.^109,110^ This and previously published work show that Oligomer materials persist within both non-wound and wound environments, where they maintain their structural and physical integrity, and exhibit physiological collagen turnover and regenerative remodeling, all of which is dependent upon the material format and implant microenvironment.^44,47,49,50^ The slow turnover observed with Oligomer materials is not surprising, since fibrillar collagen in normal connective tissues is naturally metabolized (turned over) at a very slow and controlled rate *via* a process that is distinct from immune-mediated bioresorption.^111,112^ In native tissues, molecules of fibrillar collagens are held together by mature intermolecular crosslink chemistries that influence tissue mechanical properties and collagen metabolism during tissue turnover and remodeling.^113,114^ As an inherent component of polymerizable oligomeric collagen, these mature, trivalent crosslink chemistries contribute to the distinct suprafibrillar self-assembly observed with these collagen building blocks, which in turn yields scaffolds with improved mechanical integrity and proteolytic resistance compared to those formed by other polymerizable collagens.^40,41^

The rate of cellularization and regenerative remodeling observed following implantation of Oligomer materials is dependent upon material geometry (*e.g.*, volume and shape), fibrillar density and microstructure, implantation microenvironment, and whether there is an associated tissue injury or void. In general, the remodeling rate increases with decreased scaffold volume, increased scaffold surface area, increased scaffold fibrillar density, and presence of tissue void/injury. The present study shows that Oligomer materials fashioned as relatively thin, high-density scaffolds show limited cellular infiltration and slow remodeling over a 60-day period when implanted subcutaneously, where there is limited tissue injury and no tissue void and limited disruption of the tissue mechanobiological continuum. Similar slow remodeling, along with immunoprotective properties, have been reported previously following subcutaneous implantation of islets (syngeneic, allogeneic, or xenogeneic) encapsulated within polymerized oligomeric collagen.^48,49^ More specifically, euglycemia was induced for beyond 90 days in streptozotocin-induced type 1 diabetic mice following injection/implantation of 250 islets encapsulated in 500 μL of oligomeric collagen (4.2 mg/cm^3^ in density) in two separate locations along their dorsum. In this case, the noninflammatory and persistent oligomeric scaffold supported and protected the foreign islets, preventing detection by the host immune system. In a separate study, thin, high-density Oligomer scaffolds, similar to those evaluated in the present study, were applied for reconstruction of a full-thickness mucosal layer defect (∼3–4 cm^2^) within a porcine hemilaryngectomy model.^47^ In that study, the thin, high-strength sheet was reported to provide immediate and suitable barrier function, with progressive regenerative remodeling occurring over an 8-week period. The newly formed mucosa was histologically similar to normal mucosa, featuring a stratified squamous epithelium supported by a vascularized and cellularized stromal layer with evidence of gland formation. Similar endogenous regeneration was reported when an Oligomer soft tissue filler formulation was evaluated in a porcine lumpectomy (breast conserving surgery) model.^50^ In this study, the *in situ* scaffold-forming collagen (∼8 mg/cm^3^ in density; 4–8 mL volume) conformed to and filled the complex surgical void, where it prevented contraction and supported breast tissue neogenesis, including adipose tissue and mammary glands and ducts over a 16-week period. Collectively, the regenerative remodeling observed with Oligomer materials to date has many similarities to processes associated with tissue development and morphogenesis, highlighting the importance of minimizing inflammation and maintaining the mechanobiological continuum between the tissue and material.

## 5. Conclusions

In summary, we have further defined, through histological and tissue-level transcriptomic assessments, the immunological response and mechanism of action of implantable Oligomer materials. To the best of our knowledge, this is the first study that documents that engineered polymeric materials that recapitulate molecular composition and ultrastructural features of stable, mature fibrillar collagen support regenerative remodeling *via* engagement of tissue homeostatic mechanisms, including cell-collagen mechanobiological and Th2/Treg signaling with minimal to no innate inflammation. The observed immune tolerance and tissue response is distinct when compared to traditional bioresorbable collagen scaffolds and non-resorbable Prolene meshes, which exhibit well-established innate inflammatory responses followed by mixed adaptive immune pathway activation. Given the complexity of cellular and molecular players and associated signaling with material implants and tissue morphogenesis, these studies are not without limitations. Future studies will continue to target integration of traditional and high-dimensional data analysis techniques, including proteomics and transcriptomics at both tissue and single-cell levels. Specifically, utilization of spatial and single-cell RNA-seq technologies would allow for the characterization of cell identities, phenotypes, and functionalities while also demonstrating the spatial arrangement of these phenotypes. These studies will bring further definition to cellular players and signaling mechanisms underlying Oligomer material regenerative remodeling response when applied to different anatomical locations and tissue microenvironments, including injury, non-injury, and specific disease conditions. Such multi-scale data, along with the use of computational tools and machine-learning approaches, will further guide the design and fabrication of Oligomer materials for various applications and patient-specific needs. Overall, this work further supports the translational potential of Oligomer (Collymer) as a next-generation engineering collagen polymer platform that can help address unmet tissue restoration and reconstruction needs and bring the burgeoning field of personalized regenerative medicine to clinical reality.

## Author contributions

R. A. M, S. V.-H. and S. B. designed the *in vivo* experiments. R. A. M. and S. B. performed the implantation procedures and post-surgical assessments. T. J. P. prepared the implants. R. A. M. prepared the explants for analysis. H. G. and Y. L. aided in the acquisition and interpretation of transcriptomic analysis. A. C. performed all histopathological analyses. S. V-H. and R. A. M. wrote the manuscript and all authors review, provided comments, and edited the manuscript.

## Conflicts of interest

S. V.-H. is the Founder and CSO of GeniPhys, a start-up company who licensed polymerizable collagen technology from Purdue Research Foundation for development and commercialization. T. J. P. serves as the GeniPhys Project Development Manager.

## Supplementary Material

BM-011-D3BM00091E-s001
