# Supplementary material for: Engineered collagen polymeric materials create noninflammatory regenerative microenvironments that avoid classical foreign body responses
Source: Biomater Sci. 2023 Mar 21;11(9):3278–96. doi: 10.1039/d3bm00091e (PMC10152923; doi:10.1039/d3bm00091e)
Supplement: BM-011-D3BM00091E-s001 [file BM-011-D3BM00091E-s001.pdf]

Electronic Supplementary Material (ESI) for Biomaterials Science.
This journal is © The Royal Society of Chemistry 2023

Supplementary Information

Engineered Collagen Polymeric Materials Create Noninflammatory Regenerative Microenvironments that Avoid Classic Foreign Body Responses

*Rachel A. Morrison, Sarah Brookes, Theodore J. Puls, Abigail Cox, Hongyu Gao, Yunlong Liu, and Sherry L. Voytik-Harbin**

*corresponding author: Dr. Sherry Voytik-Harbin (harbins@purdue.edu)

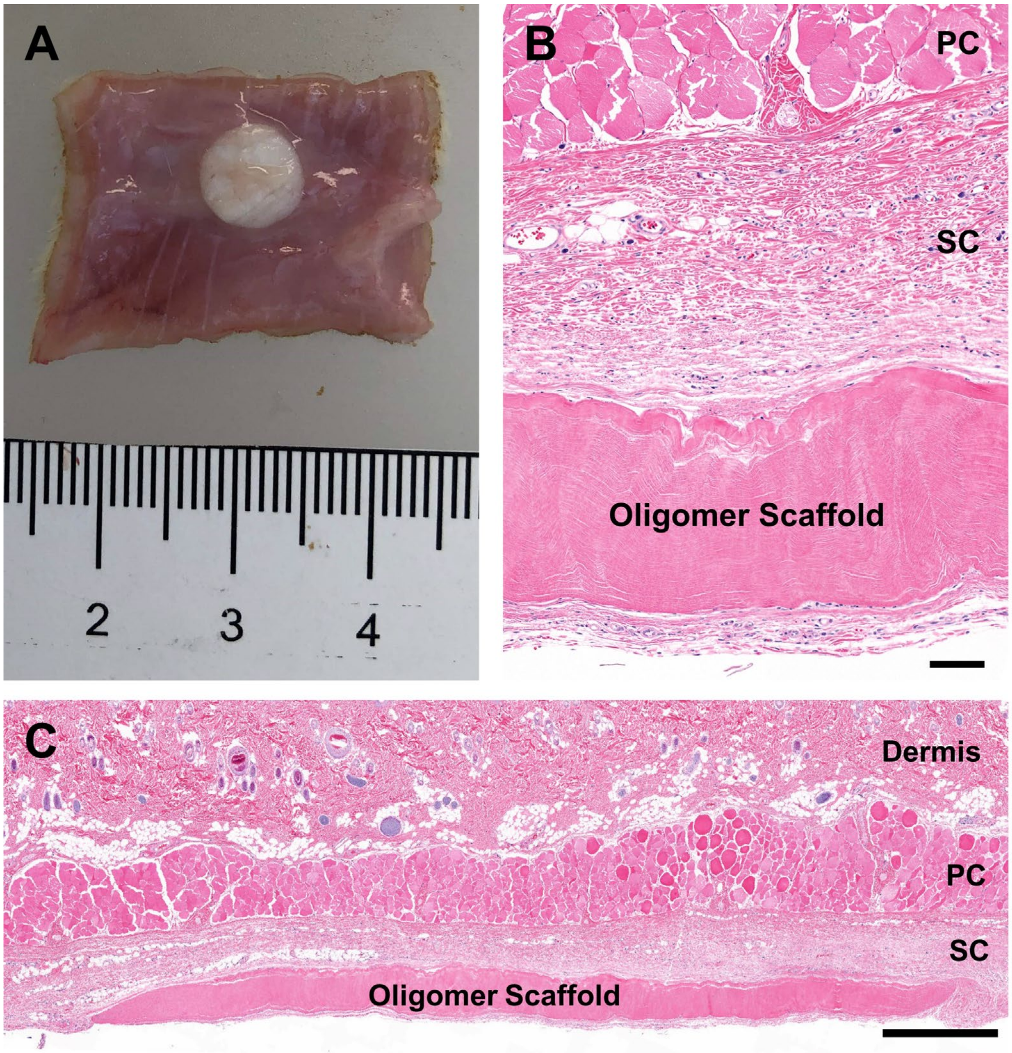

Figure S1. Oligomer scaffolds shows prolonged biocompatibility with no evidence of chronic inflammation at 60 days. (A) Images of excised Oligomer scaffolds with surrounding tissue margin. (B-C) Cross-sections (H&E) of Oligomer scaffolds. Scale bars: (B) 100µm; (C) 1mm. PC: panniculus carnosus muscle; SC: subcutaneous connective tissue.

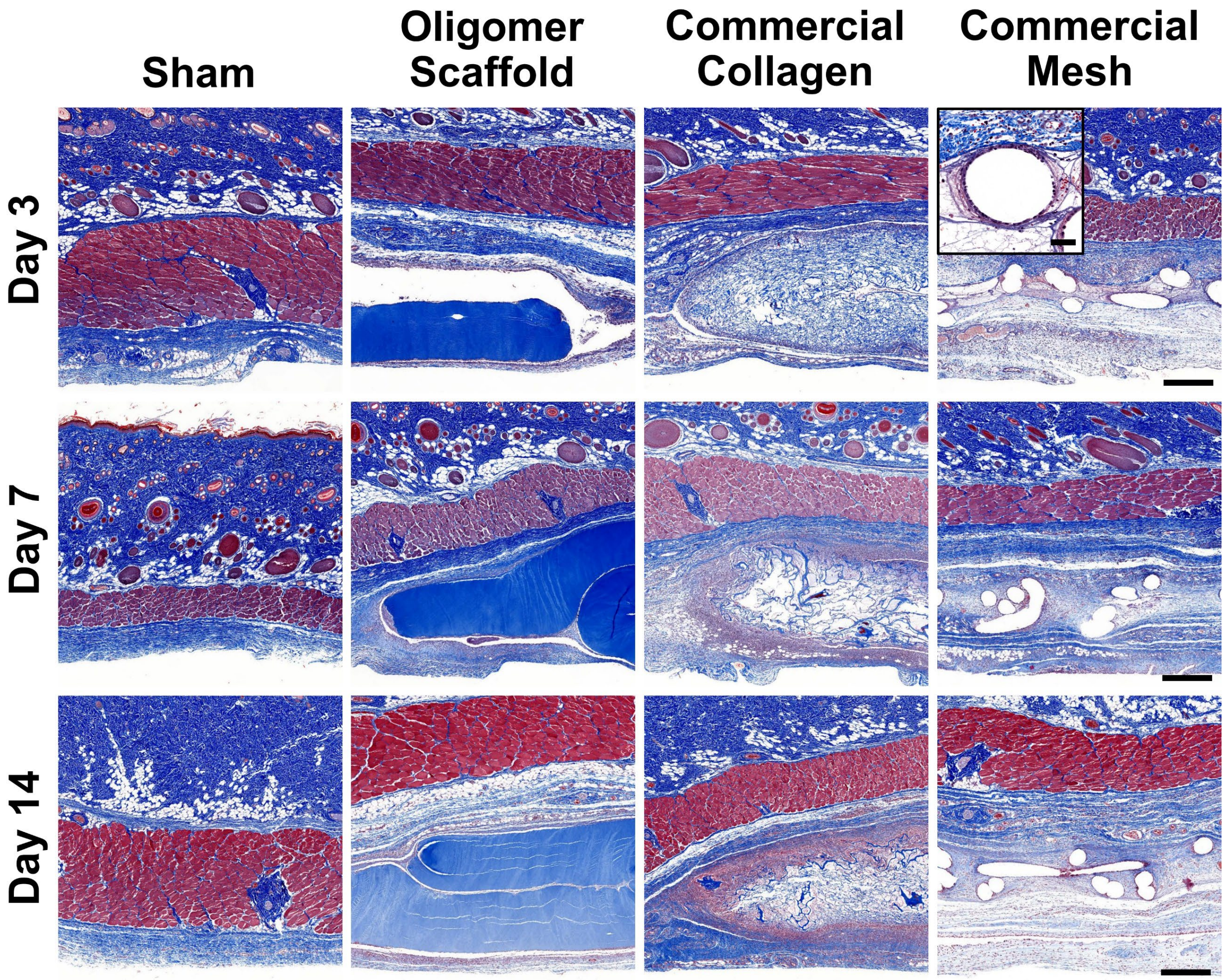

Figure S2. Cross-sections (MTC) of sham surgical site and explanted materials with surrounding tissue margin at 3-day, 7-day, and 14-day time points. Scale bars: 500 μm; inset: 50 μm

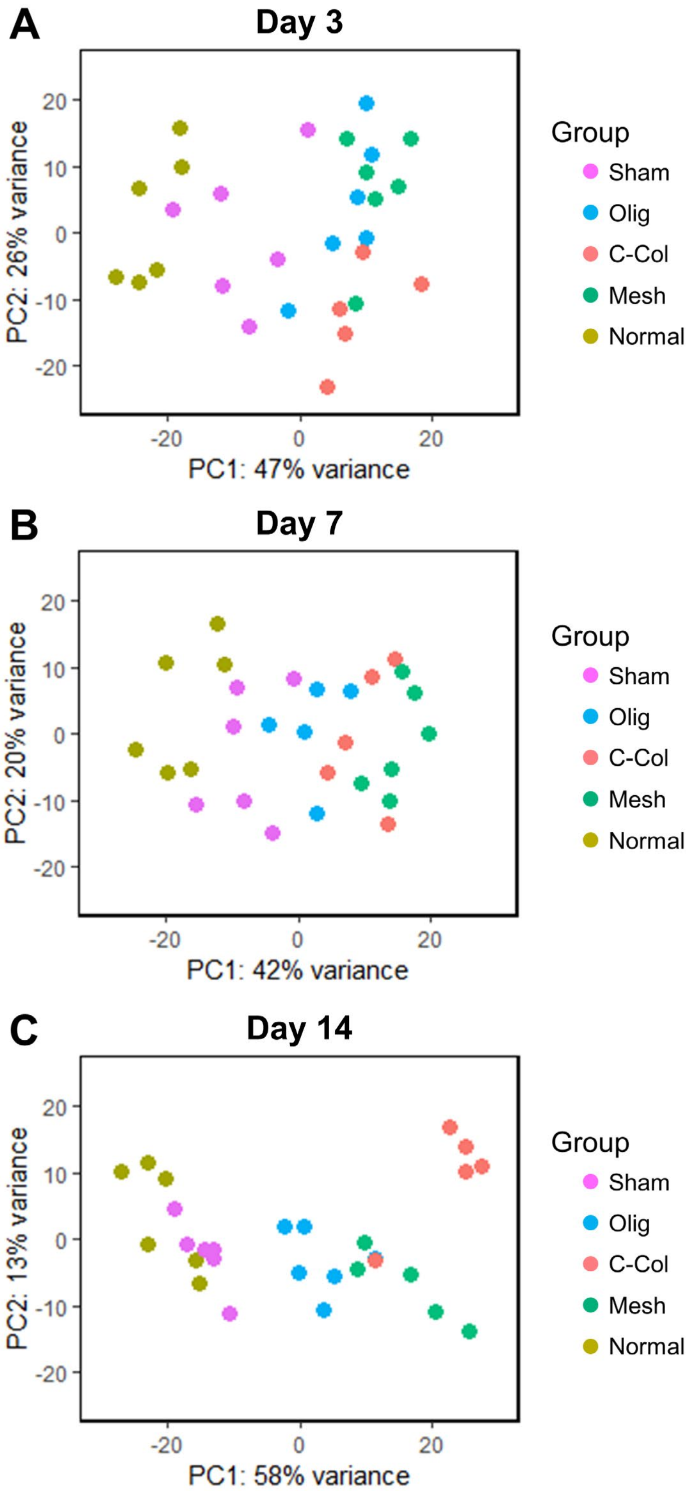

Figure S3. PCA suggests Oligomer has a different temporal molecular-level tissue response compared to conventional implant materials. PCA of RNA-seq data comparing sham, Oligomer scaffold (Olig), commercial collagen (C-Col), and mesh groups at (A) 3-day, (B) 7-day, and (C) 14-day time points relative to normal tissue.
